# Toward accurate high-throughput SNP genotyping in the presence of inherited copy number variation

**DOI:** 10.1186/1471-2164-8-211

**Published:** 2007-07-03

**Authors:** Laura E MacConaill, Micheala A Aldred, Xincheng Lu, Thomas LaFramboise

**Affiliations:** 1 Dana-Farber Cancer Institute, 44 Binney Street, Boston, Massachusetts 02116, USA; 2 The Broad Institute of Harvard and MIT, 7 Cambridge Center, Cambridge, Massachusetts 02141, USA; 3 Genomic Medicine Institute, Lerner Research Institute, Cleveland Clinic, 9500 Euclid Avenue, Cleveland Ohio 44195, USA; 4 Department of Genetics, Case Western Reserve University, 10900 Euclid Avenue, Cleveland Ohio 44106, USA

## Abstract

**Background:**

The recent discovery of widespread copy number variation in humans has forced a shift away from the assumption of two copies per locus per cell throughout the autosomal genome. In particular, a SNP site can no longer always be accurately assigned one of three genotypes in an individual. In the presence of copy number variability, the individual may theoretically harbor any number of copies of each of the two SNP alleles.

**Results:**

To address this issue, we have developed a method to infer a "generalized genotype" from raw SNP microarray data. Here we apply our approach to data from 48 individuals and uncover thousands of aberrant SNPs, most in regions that were previously unreported as copy number variants. We show that our allele-specific copy numbers follow Mendelian inheritance patterns that would be obscured in the absence of SNP allele information. The interplay between duplication and point mutation in our data shed light on the relative frequencies of these events in human history, showing that at least some of the duplication events were recurrent.

**Conclusion:**

This new multi-allelic view of SNPs has a complicated role in disease association studies, and further work will be necessary in order to accurately assess its importance. Software to perform generalized genotyping from SNP array data is freely available online [1].

## Background

A copy number variant (CNV) is defined as a chromosomal segment, at least 1 kb in length, whose (germline) copy number varies across individuals in the human population [2]. As the importance of these duplications and deletions in the study of a variety of diseases [3-6] is being realized, cataloging them and assessing their frequencies has become an important goal. Toward this end, two recent studies [7,8] have exploited erroneous SNP genotype calls, inferring germline deletions at clusters of calls that violate Mendelian inheritance or other conditions. The violations occur, however, as a result of the (diallelic) assumption of three possible genotypes (*e.g*. GG, GT, or TT) at each SNP site. If this assumption of two copies at each locus were relaxed, one could consider a generalized genotype whereby the SNP is multi-allelic when considering both base residue and copy number. An individual could carry, for example, a GGT (duplication), G – (hemizygous deletion), or – (homozygous deletion) genotype at a SNP locus. As most recent estimates put the proportion of the genome harboring CNVs at at least 12% [9], allowing for more general genotypes is crucial for the accuracy of SNP typing in disease studies. Such direct and accurate typing would, of course, reveal CNVs automatically.

The GeneChip Human Mapping Array Set [10] is a popular platform for high throughput SNP genotyping. We use data from the version of the platform – herein referred to as the SNP array – that interrogates over 500,000 SNP sites. Since 85% of the genome is within 10 kb of at least one SNP on the array [10], many of the duplications and deletions of the size that have been reported thus far should contain several of the SNP sites represented on the array. Indeed, 58,353 of its 490,032 autosomal SNPs are contained in at least one of the CNVs that have been reported in the literature to date and catalogued in the Database of Genomic Variants [11]. In an earlier study [12] we utilized the array data to detect, in an allele-specific manner, somatic copy number changes in cancer samples, also demonstrating an extremely high genotyping accuracy (> 99.7%) in the diallelic setting. We therefore endeavored in the present study to adapt this approach to SNP array data from "phenotypically normal" individuals in an effort to provide a generalized genotype, allowing for germline CNVs as described above. The approach is somewhat akin to array CGH [13] methods to detect CNVs, but with at least two advantages. First, it is difficult in array CGH analysis to determine whether an apparent deviation from copy number two is the result of a CNV in the test sample or in the reference sample. In our approach, we exploit a large reference panel of individuals, ensuring that the reference signal is essentially two-copy, except potentially in regions with very common CNVs. Second, while the array CGH platforms lack allele-specific information, the SNP array is comprised of oligonucleotide probes that can distinguish between each of the two alleles of each SNP. Our method models these probes' intensities as a function of allele-specific copy number, which directly determines the generalized genotype. The copy numbers are inferred from the SNP array data by applying statistical model-fitting procedures.

## Results and discussion

### Detected aberrations

**Figure 1:**
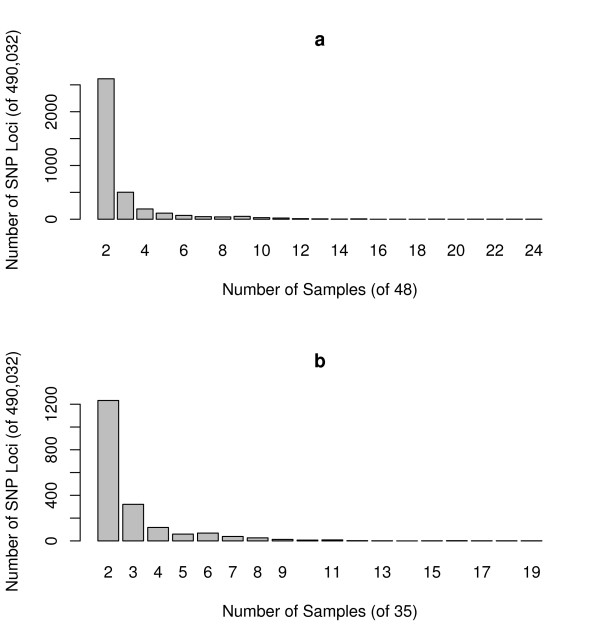
**Frequencies of aberrant SNPs in our study**. For each SNP, the number of samples aberrant at that locus was counted. We constructed histograms of frequencies for SNPs that were aberrant in more than one sample. (*a*) For each count *c *on the horizontal axis, the height of the bar indicates the number of SNPs that were aberrant in *c *samples out of 48. (*b*) Same as *a*, but with all trio offspring removed so that only 35 unrelated samples are considered.

We analyzed SNP array data from a collection of 48 individuals of various ethnic backgrounds. For a reference panel, we used 16 unrelated individuals of African, African-American, Asian, European, and Hispanic ethnicities (see Methods). An ethnically diverse panel of reference samples minimizes the likelihood of a recurrent CNV skewing the "copy number two" reference signal. Applying our algorithm to all automsomal SNPs on the array, we found 21,568 SNP loci that demonstrated aberrant genotypes (Additional data file 1). Of these, 17,390 were detected as duplications (total copy number > 2), 5,051 as hemizygous deletions (total copy number 1), and 214 as homozygous deletions (total copy number 0). There were 881 sites that were detected as both duplications and deletions. The 21,568 SNPs can be grouped into 5,622 regions of consecutive duplicated SNPs and 1,130 regions of consecutive deleted SNPs Regarding recurrence, 3,721 (17.3%) SNPs were aberrant in more than one individual (Figure 1a), with one SNP (rs1842908) showing a non-diploid genotype in 24 of the 48 individuals.

### Experimental validation

**Figure 2:**
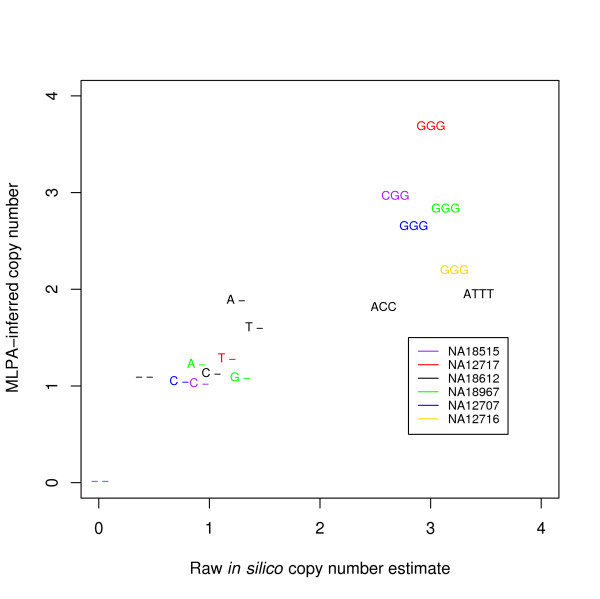
**Concordance between *in silico *and MLPA results**. We plotted the MLPA-inferred dosage against our raw total copy number inferences (see Methods) from the SNP array data. The plotting symbol for each SNP site is its genotype inferred by our procedure, and the color indicates the sample. The concordance is very strong with the exception of one sample, NA18612 (black), which could be due to either noisy array data or experimental difficulties for that particluar sample.

In order to validate our discoveries using independent experimental means, we performed quantitative real-time PCR (qPCR) experiments for 30 of the regions containing putatively aberrant SNPs, using DNA from individuals in our sample set. A comparison of the qPCR results with our genotype inferences is given in Table 1. Overall, 26 (86.7%) of the qPCR results agreed with the presence and type (duplication or deletion) of CNV predicted by our *in silico *approach. There are a variety of potential reasons for the four nonconcordant loci. Since the PCR primers are explicitly designed to avoid the SNP sites (yet be near the aberrant SNPs), and since CNVs can be quite focal, it is possible for the PCR-amplified region to miss the aberrant locus entirely. A detected deletion could also be an artifact of the SNP array assay, since the deletion or duplication elsewhere in the restriction fragment may result in its length moving outside the range of lengths that the assay's PCR step would amplify [10]. As mentioned above, common CNVs in the human population are another potential source of error, since it is implicitly assumed that the reference panel primarily harbors two copies per cell at each locus. To provide further independent validation, we also performed multiplex ligation-dependent probe amplification (MLPA) [14] experiments on 17 other putative CNVs (Figure 2 and Additional data file 2). The concordance with the *in silico *genotypes was similar to that of the qPCR results.

**Table 1:** Comparison of *in silico *and *in vitro *results for 30 putatively aberrant SNPs. Here, the diploid genotype refers to that provided by the SNP array's default software [24], under the assumption of two copies of the SNP. The errors shown are typical in the presence of CNVs. The aberrant genotype here is our algorithm's call. We consider the putative CNV to be validated if the rounded qPCR copy number is less than 2 (for deletions) or greater than 2 (for duplications).

Sample	rs ID	Diploid genotype	Aberrant genotype	Frequency (unrelated samples)	qPCR copy number	Validated?
NA10851	rs17525374	GG	G -	1	1.17	Yes
NA10851	rs9542207	TT	T -	1	0.86	Yes
NA10851	rs6601728	GG	G -	1	1.40	Yes
NA10851	rs17133566	CC	C -	1	0.98	Yes
NA10863	rs5751296	CT	CTT	1	2.74	Yes
NA10863	rs17577094	TT	TTT	5	4.27	Yes
NA12707	rs1565516	GG	G -	1	1.95	No
NA12707	rs2013317	GG	GGG	4	6.14	Yes
NA12801	rs2304717	CC	CCC	1	4.29	Yes
NA12801	rs12697975	CC	C -	1	0.84	Yes
NA12801	rs2889833	GG	G -	1	1.24	Yes
NA10851	rs11780672	AA	A -	1	0.76	Yes
NA10863	rs3828886	AA	AAA	8	2.12	No
NA10863	rs3858489	AG	AGG	5	3.40	Yes
NA10863	rs17662235	TT	TTT	7	3.36	Yes
NA10863	rs6994627	AA	A -	1	0.88	Yes
NA10863	rs2604357	GG	G -	1	1.54	No
NA10863	rs10110189	CC	C -	1	0.85	Yes
NA10863	rs2721243	CC	C -	1	1.01	Yes
NA10863	rs737714	No Call	A -	1	0.00	Yes
NA10863	rs7833963	AA	A -	1	1.05	Yes
NA10863	rs4831667	AA	A -	1	0.82	Yes
NA10863	rs7464441	AA	A -	1	1.36	Yes
NA12707	rs1018685	AA	AAA	1	1.59	No
NA12707	rs16993280	No Call	- -	3	0.00	Yes
NA12707	rs361901	AA	A -	4	1.40	Yes
NA12707	rs12170791	CC	C -	2	0.98	Yes
NA12707	rs2532292	AA	AAA	7	4.51	Yes
NA12707	rs2732675	No Call	TTT	10	2.53	Yes
NA12707	rs4822622	CC	C -	1	1.06	Yes

### Mendelian inheritance considerations

**Figure 3:**
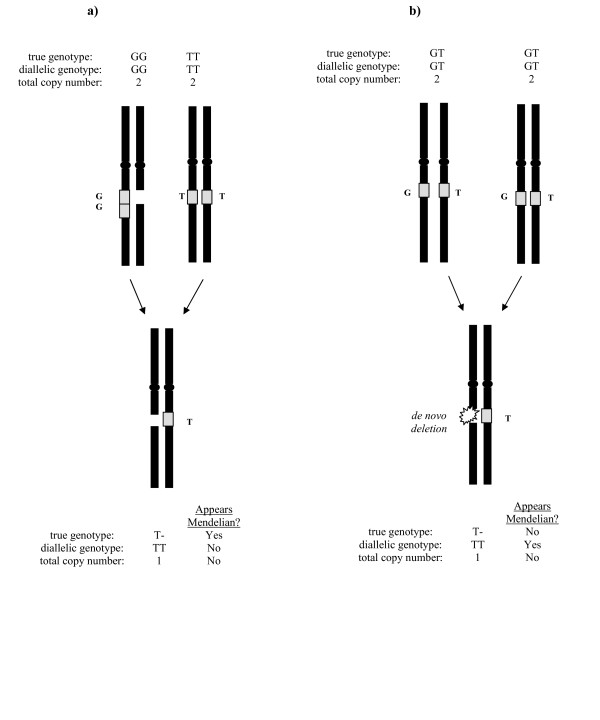
**Mendelian and non-Mendelian inheritance patterns in the presence of CNVs**. (*a*) Although the parent on the left has copy number two at the locus, this is the result of a duplication on one chromsome and a deletion on the other. Standard (diallelic) genotyping methods would incorrectly identify a non-Mendelian pattern at the locus in this trio, as would total copy number information alone. However, accurate genotyping, taking CNVs into account, reveals that the pattern is Mendelian. (*b*) The *de novo *deletion is obscured when diallelic genotyping alone is considered, though copy number information reveals the event.

It is well-established [9,15,16] that there are genomic regions harboring germline CNVs in the form of both duplications and deletions in different individuals. Indeed, the non-allelic homologous recombination model of the formation of the variants generates both duplications and deletions, simultaneously at the same locus. It follows that, as these variants segregate through the population, there will be individuals carrying both a gain and a loss at the same locus, though on different parental chromosomes. The observable patterns of Mendelian inheritance in the aberrant genotype setting are different from those in diallelic SNP genotyping or even in aggregate copy number measurement, and ignoring the presence of CNVs can result in the false appearance of non-Mendelian inheritance as a result of genotyping errors (Figure 3a and Additional data file 3). Truly non-Mendelian inheritance, *e.g. de novo *events (Figure 3b and Additional data file 3) can be masked as well. These errors will also occur when only aggregate copy number is measured but allelic information is ignored. Misinterpreting germline CNVs as *de novo*, or *vice versa*, can have important implications, particularly in clinical settings where such variants in an affected individual are considered less relevant when inherited from an unaffected parent [17]. With multi-allelic SNP genotypes, we should be able to distinguish between the two cases. We were able to check aberrant SNPs for Mendelian inheritance in the 13 mother-father-child trios in our data set. Accounting for both SNP and copy number variation, 1,535 of the 1,771 instances of putatively aberrant SNPs in the four individuals (86.7%) demonstrated Mendelian inheritance. Possible explanations for non-Mendelian events include *de novo *CNVs or uniparental disomy, both of which have been detected using the SNP array platform [18-20]. Alternatively, detected CNVs may be instead artifacts of cell line culture, as observed in [9].

### SNP alleles in duplications

**Figure 4:**
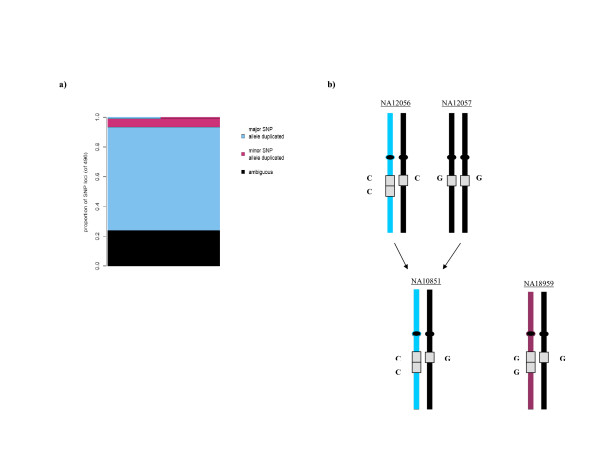
**Interplay between duplication status and SNP allele**. (*a*) Of 496 interrogated duplication loci, we observed 6 cases (1.2%) with both AA and BB chromosomes, and none with AB chromosomes. When only one SNP allele was unambiguously duplicated, 92.5% of the time it was the major allele in our sample set. (*b*). An example of a Caucasian trio and a Japanese individual harboring different SNP alleles in the duplicon.

Examination of the SNP allelic composition of duplicated regions can provide insight into the history of copy number variation in the human population. In the case of a SNP site that is duplicated with (haploid) copy number two, there are theoretically five possible haploid genotypes for that SNP: AA, AB, BB, A, and B, where A and B are the two base residues for the SNP site. The presence or absence of each of these five haploid genotypes sheds light on recurrence and temporal order of both the duplication event at that locus as well as the point mutation that resulted in the SNP. For example, the presence of both AA and BB would imply a recurrent duplication, occurring on both the A and B SNP background. To investigate empirically, we examined 496 aberrant loci in detail (see Methods). We found no evidence of a single chromosome with different base residues on each copy of a duplication (i.e. an AB chromosome) (Figure 4a). It is therefore unlikely that the "SNP in duplicon" phenomenon noted recently [21] for segmental duplications is common in CNVs. This is also consistent with the conclusion of the HapMap consortium [22] that the point mutations leading to SNPs are largely non-recurrent. In the vast majority of cases, only one of the SNP alleles was duplicated in our sample set. Still, six (1.2%) of the SNP sites had evidence of both AA and BB chromosomes. The presence of both types show that at least some duplication events were most likely recurrent in human history (alternative explanations seem unlikely, particularly given the complete absence of evidence for any AB chromosomes). An example is rs7895458 on chromosome 10 (Figure 4b), which is contained in a previously known recurrently duplicated region [9]. In our data set, one Caucasian family – NA12056 (father) and NA10851 (child) – harbors the duplication with the C allele at the SNP in both copies. Japanese individual NA18959, on the other hand, harbors the duplication with the G allele at the SNP in both copies. Interestingly, the dbSNP database [23] lists the allele frequency for C as 65% in the Caucasian population, but only 15% for the Japanese population. These duplications were observed independently in [9] in all three of these individuals, though the SNP allelic information was ignored in that study. Similarly, the SNP array manufacturer's [24] diallelic calls, CC for NA10851 and GG for NA18959, were erroneous though consistent with what was expected given their two-copy assumption. This example points to the insights that can be gained from consideration of both copy number and SNP residue simultaneously.

### Comparison with previous work

Since our detected aberrant SNPs automatically indicate the presence of CNVs, we compared the genomic coordinates of these loci to CNVs previously reported in the literature. Of our 21,568 SNP loci, 5537 (25.7%) are contained in regions catalogued as CNVs in the Database of Genomic Variants, while only 11.9% of the 490,032 autosomal SNPs on the SNP array are contained in these regions. Though this demonstrates more overlap than would be expected by chance, the majority of loci we have uncovered are novel. However, one would expect that recurrently variant SNPs in our data set would often exist at a higher frequency in the general population, and would thus be more likely to have been previously discovered. Indeed, if we restrict our attention to SNPs that are aberrant in at least two unrelated individuals in our sample set, we find that 1062 of 1905 (55.7%) of these lie in previously reported CNVs. As the aberrance rate of a SNP in our sample increases, its likelihood of having been previously reported continues to rise – over 95% of the 351 SNPs that are aberrant in at least 10% of our unrelated sample set are contained in previously reported CNVs. Therefore, although we report thousands of new CNVs, our results are in some sense concordant with what has been revealed on a population level. Since it has been shown that sequence between intrachromosomal segmental duplications are predisposed to CNVs [25], we also checked for enrichment of these "hotspots" [16] in our set of CNVs. Only 2,336 (10.8%) of our aberrant SNP loci were contained in these regions reported in the Structural Variation Database, which is only a slightly higher proportion than that of all autosomal SNPs on the array (8.1%).

### Genes in regions of copy number variation

Many genes previously confirmed as polymorphic have functions in metabolism and immunity, and likely act as mediators of normal human variation as well as genomic disease. We compiled a list of transcribed CNVs in our data, along with the Gene Ontology [26] (GO) terms that were associated to these transcripts. We examined our list for GO terms that were present at a statistically higher rate compared to all genes containing SNPs represented on the array (see Methods). This allowed the identification of several interesting categories of genes involved in cell surface structure, glutamate metabolism and signaling, and genes with metabolic, enzymatic and neurological functions. In concordance with previous studies, we confirmed the presence of CNVs in genes such as DUSP22, NCAM2 [27], and NF1 [16]. Also present in our list are genes that are known to influence "normal" human phenotypes, such as the copy number-polymorphic olfactory receptor genes [28], and the neuropeptide-Y4 receptor PPYR1 [27] that is directly involved in the regulation of food intake and body weight [29]. A number of "environmental sensor" genes involved in immune system response were also observed, in categories such as granulocyte differentiation, receptor-mediated endocytosis, antibiotic response, regulation of IgG/IgE isotype switching, regulation of NK cell activity, IL-4 receptor binding and MHC class 1 receptor activity. In addition, a large proportion of the CNV-enriched classes have receptor and/or signaling functions. It is important to note that a number of genes previously reported to be copy number variant, such as the glutathione S-transferase genes GSTM1 and GSTT1, are not represented on the SNP array, perhaps due to the fact that they would give ambiguous genotype calls.

## Conclusion

We have presented the first computational method for genotyping SNPs from microarray data in the general case where the individual is not restricted to two copies of the SNP locus per cell. Our work highlights, with several examples, the relevance of considering both copy number and SNP allelic information simultaneously. We have uncovered tens of thousands of SNPs with aberrant genotypes in humans of various ethnicities, corresponding to thousands of novel CNVs. It is likely that our results actually drastically underrepresent the prevalence of aberrant SNPs in the population, as the array's manufacturer deliberately excluded SNPs that violated Hardy-Weinberg equilibrium, Mendelian inheritance, and other quality control requirements [10] that would naturally not be met in the presence of CNVs. Moreover, our own requirement that at least three consecutive SNPs show the CNV is very conservative, and will by definition omit more focal events (in practice, our method can be tuned in this way to control the false positive/false negative tradeoff, as desired). It follows that the number and frequency of these multi-allelic SNPs segregating through the population is likely to be much more substantial than previously suspected, and therefore generalized genotyping of the sort we have described here is crucial in studies using SNPs as markers. Our work is a step in that direction, though the goal should be to attain the high rates of accuracy (> 99%) that are assumed in the diallelic setting. Such highly accurate genotyping would automatically provide information regarding the presence or absence of CNVs. Given the difficulty in precisely mapping the boundaries of these germline CNVs (with recent evidence that the boundaries actually differ between individuals in the population [30]), and given the density of SNPs on the genome, we propose aberrant SNP genotyping as an alternative to other methods of categorizing CNVs from SNP array data [31]. Such genotypes map to precise genomic locations, and provide information on both copy number and base residue. The resulting multi-allelicism will be an aid to disease association studies, whether these more accurately ascertained SNP alleles are actually causal inherited variants, or are simply used as markers. Since there are many thousands of SNP array samples extant, these multi-allelic SNPs segregating through the population will be identified and their frequencies ascertained. We have developed software, freely available at our web site, with which users can scan their arrays for aberrant SNPs, using reference panels of their choice. As the platforms increase in throughput and decrease in cost, accurate multi-allelic genotyping will be even more crucial.

## Methods

### SNP array data and biological samples

The raw .cel files from the 48 individuals – NA10851, NA10855, NA10863, NA11831, NA11832, NA12056, NA12057, NA12234, NA12264, NA12707, NA12716, NA12717, NA12801, NA12812, NA12813, NA18503, NA18504, NA18505, NA18506, NA18507, NA18508, NA18515, NA18516, NA18517, NA18532, NA18545, NA18558, NA18605, NA18612, NA18959, NA18967, NA18969, NA18997, NA19137, NA19138, NA19139, NA19152, NA19153, NA19154, NE00088, NE00090, NE00091, NE00375, NE00403, NE00598, NE00963, NE01118, and NE01119 – in the Mapping 500 K Sample Data Set were downloaded from the Affymetrix web site [32]. These individuals are of African (15), European American (15), Han Chinese (5), Japanese (4), African American (3), Asian American (3), and Hispanic American (3) ethnicities. DNA and cell lines from 20 of these individuals was obtained from the Coriell Cell Repositories for qPCR and MLPA experiments.

### Generalized genotyping and candidate CNVs

We used 16 individuals – NA11831, NA12057, NA18505, NA18507, NA18517, NA18532, NA18545, NA18558, NA18959, NA18967, NA18969, NA19138, NA19152, NE00090, NE00403, and NE01119 – as our reference panel, selected because they are unrelated and are from a variety of ethnic backgrounds. Using the data from these 16, we trained the PLASQ [12] model parameters as described. We then used PLASQ to infer the "raw" allele-specific copy numbers (ASCNs) in our test samples, restricting our attention to the autosomes. The pairwise sums of the raw ASCNs yielded raw total copy numbers, which were rounded to the nearest integer for total copy numbers. Calls with total copy number deviating from two provided a preliminary list of aberrant SNPs. These were converted to the generalized genotypes by assigning the whole-number portions of the total copy number to each allele so that the (nearest integer) raw ASCNs were retained as much as possible. In order to enrich our candidate set for true positives, we restricted our attention to SNPs in runs of at least three independent aberrant SNPs with aberrations in the same direction (duplication or deletion). In this context, we consider adjacent SNPs to be independent only if they reside on different restriction enzyme fragments, since fragment-specific artifacts arising during the PCR step of the SNP array protocol [10] would presumably affect all SNPs on the fragment.

### PCR validation of CNVs

Relative gene copy numbers were determined by quantitative real-time PCR using a PRISM 7900HT Sequence Detection System (384 well) (Applied Biosystems, Foster City, CA). Real-time PCR was performed in 12.5-*μ*l (384 well) reactions with 2 ng of template DNA. A QuantiTect SYBR Green PCR kit (Qiagen Inc., Valencia, CA) was used for the PCR reaction. PCR conditions were as follows: 2 min at 50°C, 15 min at 95°C, followed by 40 three-step cycles of (20 s at 95°C, 20 s at 58°C, and 30 s at 72°C).

Primers were designed using Primer 3 [33] and synthesized by Integrated DNA Technologies (IDT; Coralville, IA). Primer sequences are available upon request. Quantification was based on standard curves from a serial dilution of human normal genomic DNA. The standard curve method was used to calculate target DNA copy number in each DNA sample normalized to a repetitive element Line-1 and normal reference DNA. For our reference sample, we used female genomic DNA pools, derived from multiple anonymous donors (Promega, Madison, WI), since combining DNA from multiple individuals should dilute out all but the most common copy number variants.

### MLPA validation of CNVs

Custom MLPA probes were designed to match suitable sequences within 300 bp of the original SNP location. Control probes were drawn from other chromosomal locations and have previously been used to analyze more than 100 individuals without evidence for copy number variation [34]. Oligonucleotides were synthesized by IDT, with 5'-phosphorylation of each downstream probe and tagged with common PCR primer sequences [14]. Probes were hybridized with 100 ng aliquots of DNA using MLPA reagents (part number EK5, MRC-Holland BV, Amsterdam, The Netherlands) according to the recommended protocol. Samples were then diluted 10-fold and analyzed on a 3730xl sequencer with GeneMapper software (Applied Biosystems). We used male and female genomic DNA pools, derived from multiple anonymous donors (Promega, Madison, WI). Furthermore, peak height ratios were normalized to the mean of the entire data set, rather than to the controls alone, with subsequent elimination of outlier samples from the calculation of the mean. Our experience with well-characterized deletions shows that this approach gives equivalent results to normalizing against controls alone, provided that samples with altered copy number are in the minority (data not shown).

### Analysis of SNP alleles in duplications

Determining which SNP alleles are harbored in a duplication is subject to the same phasing difficulties as SNP haplotype determination. In order to maximize our ability to determine correct phasing, we considered only SNP sites that were duplicated to total copy number three in at least one trio offspring, with one parent having copy number three and the other either two or three. To avoid the possibility of a deletion on one chromosome, we omitted from consideration all loci contained in a deleted regions in any individual (either in our data or in the Database of Genomic Variants). This left us with 496 SNPs for which we sought to detect the presence of the AA, AB, BB, A, and B chromosomes in our sample set, through a combination of phasing and examination of individual genotypes (see Additional data file 4). Note that lack of detection does not necessarily indicate absence, but could instead be the result of ambiguous phase.

### Comparison with previously-published CNVs and segmental duplications

The previously published CNVs were those catalogued (as of December 4, 2006) in the Database of Genomic Variants (Build 35 coordinates). The "rearrangement hotspots" [16] are regions, between 50 kb and 10 Mb in length, flanked by segmental duplications at least 10 kb in length with at least 95% sequence identity. The Build 35 coordinates of these segmental duplications were downloaded from the Segmental Duplication Database [35] on December 4, 2006.

### Statistical analysis of GO associations in CNVs

We mapped all SNPs on the array to their genomic positions using the UCSC Genome Browser (Build 35). The 11,944 genes whose transcribed regions contain at least one of the (autosomal) SNPs on the array comprised our "gene universe". Our duplicated genes and deleted genes were those with transcripts containing SNP sites that were duplicated or deleted, respectively, in at least one of our 48 samples. We made use of the R [36] software package GOstats [37] to test our duplicated and deleted genes for statistical enrichment in certain GO terms (as annotated by the hgu133plus2 package) [38]. Briefly, for a fixed GO term, the software performs a Fisher's exact test for the null hypothesis of no association between duplication or deletion status and annotation to that term, using all genes in our gene universe.

## Authors' contributions

LEM carried out all PCR experiments, helped refine the computational method, and drafted parts of the manuscript. MAA carried out all MLPA experiments and drafted parts of the manuscript. XL contributed expertise in the handling and extraction of DNA. TL conceived of the study, designed the statistical methodology, and wrote the manuscript. All authors read and approved the final manuscript.

## Supplementary Material

Additional file 1 **All 21568 duplicated and deleted SNPs**. This table lists all 21568 detected SNPs. Included in the table are SNP ID, genomic coordinates, frequency of deletion/duplication, and the gene harboring the SNP. Click here for file

Additional file 2 **Comparison of in silico results with MLPA results**. This table lists sample, SNP ID, genomic coordinates, MLPA copy number, and in silico copy number and generalized genotype for all 17 SNP loci that were compared. Click here for file

Additional file 3 **Mendelian and non-Mendelian inheritance patterns identified by generalized genotype**. This figure shows two hypothetical cases in which the generalized genotype accurately assesses Mendelian inheritance. a) Under the assumption of a diallelic genotype, the inheritance appears to be non-Mendelian. When copy number variation is taken into account, Mendelian inheritance is revealed. b) The *de novo *duplication is obscured when total copy number alone is considered. However, the true genotype uncovers this event, since allelic information is taken into account. Click here for file

Additional file 4 **Supplementary Methods**. This file describes how we were able to "phase" the alleles in duplicated SNPs. Click here for file
