# Supplementary material for: Toward accurate high-throughput SNP genotyping in the presence of inherited copy number variation
Source: BMC Genomics. 2007 Jul 3;8:211. doi: 10.1186/1471-2164-8-211 (PMC1934372; doi:10.1186/1471-2164-8-211)
Supplement: Additional file 3 — Mendelian and non-Mendelian inheritance patterns identified by generalized genotype. This figure shows two hypothetical cases in which the generalized genotype accurately assesses Mendelian inheritance. a) Under the assumption of a diallelic genotype, the inheritance appears to be non-Mendelian. When copy number variation is taken into account, Mendelian inheritance is revealed. b) The de novo duplication is obscured when total copy number alone is considered. However, the true genotype uncovers this event, since allelic information is taken into account. [file 1471-2164-8-211-S3.pdf]

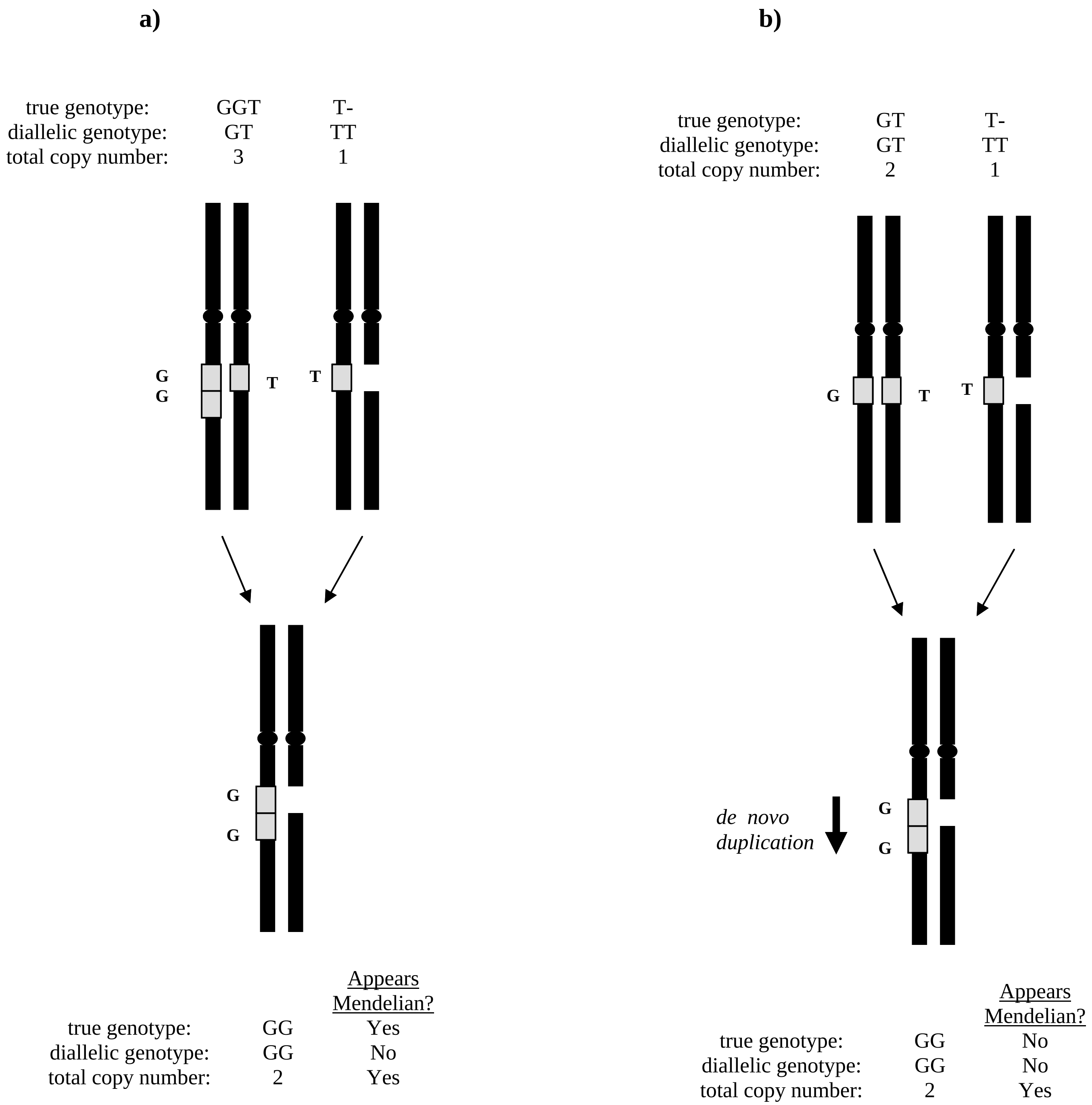
a)
true genotype: GGT T-
diallelic genotype: GT TT
total copy number: 3 1
G
G
T
T
G
G
Appears Mendelian?
true genotype: GG Yes
diallelic genotype: GG No
total copy number: 2 Yes
b)
true genotype: GT T-
diallelic genotype: GT TT
total copy number: 2 1
G
T
T
de novo duplication
G
G
Appears Mendelian?
true genotype: GG No
diallelic genotype: GG No
total copy number: 2 Yes
