# Supplementary material for: Toward accurate high-throughput SNP genotyping in the presence of inherited copy number variation
Source: BMC Genomics. 2007 Jul 3;8:211. doi: 10.1186/1471-2164-8-211 (PMC1934372; doi:10.1186/1471-2164-8-211)
Supplement: Additional file 4 — Supplementary Methods. This file describes how we were able to "phase" the alleles in duplicated SNPs. [file 1471-2164-8-211-S4.pdf]

Supplementary Methods

In order to simplify our analysis of duplications and point mutations to make inferences about the history of these events in humans, we restricted our attention to SNP that:

a) are among the 21,568 “high confidence” events
b) are copy number three in at least one of the 13 trio offspring
c) are copy number three in at least one of these individuals’ parents
d) in at least one of the pairs satisfying b) and c), the other parent has copy two or three at this SNP
e) have is no evidence for a germline deletion of the SNP, either in our samples or in the Database of Genomic Variants

In this way, we can guarantee that the copy-number-three parent has genotype AAA or AAB (assigning A to the major allele, without loss of generality). Condition e) avoids the possibility of a deletion on one chromosome and a duplication on the other.

There were a total of 496 SNPs satisfying all of these criteria. For each SNP site, we used the following procedure to determine the presence or absence of AA, AB, A, BB, and B chromosomes. First, diploid genotypes of AA or AB anywhere in our sample set guaranteed the presence of an A chromosome, while AB or BB diploid genotype guaranteed the presence of a B chromosomes. Similarly, any AAA or BBB genotypes guaranteed the presence of AA or BB chromosomes, respectively.

To infer anything further, phasing information from copy number three AAB individuals is necessary (to distinguish AA, AB, and BB chromosomes). If both parents are homozygotes, no new information is gained. If, however, one parent is AAB, one can sometimes infer phasing according to the following table. The rows here represent the genotype of the other parent, and the columns represent the offspring. The entries indicate the chromosomes that are guaranteed to be present under the corresponding trio genotype combination. A blank entry indicates a non-Mendelian inheritance pattern.

2nd parent\child	AAA	AAB	ABB	BBB	AA	AB	BB
AAA	AA, A	AA, A, B			AA, AB, A	AA, A, B	
AAB	AA, AB,A, B	AB	AA, AB, A, B		AB, A	AA, AB, A, B	AA, B
ABB	AA, BB, A, B	AB	AB	AA, A, BB, B	AB, A, BB	A, B	AA, AB, B
BBB		AA, BB, B		AA, BB, B		AB, A, BB, B	AA, BB, B
AA	AA, A, B	AB, A			AB, A	AA, A, B	
AB	AA, A, B	A, B	AB, A, B		AB, A, B	A, B	AA, A, B
BB		AA, B	AB, A, B			AB, A, B	AA, B

In this way, we obtain a list of all observed haploid genotypes (out of the five possible) at the given SNP, with the understanding that our not observing a genotype does not guarantee its absence in the human population, or even in our sample set.
